# How Bilingual Parents Talk to Children About Number in Mandarin and English

**DOI:** 10.3389/fpsyg.2019.01090

**Published:** 2019-05-14

**Authors:** Alicia Chang, Catherine M. Sandhofer

**Affiliations:** Department of Psychology, University of California, Los Angeles, Los Angeles, CA, United States

**Keywords:** bilingual, number words, language input, children, cardinality

## Abstract

Number-related language input has been shown to influence children’s number word acquisition and mathematical ability. Significant differences exist between how Mandarin Chinese speaking parents and monolingual English-speaking parents use numeric language in speech to children. In particular, Mandarin Chinese speaking parents use cardinal number much more frequently in speech to children than do English speaking parents. However, because previous studies have been conducted cross-nationally, research has been unable to disentangle the influences of language from parental influence. The current study examined numeric language input to preschool children with bilingual Mandarin-English American parents. Results show that when parents speak to their children in Mandarin Chinese, children hear more instances and examples of the cardinal number principle than when parents speak to their children in English. This suggests that differences between how the Mandarin Chinese and English languages are structured leads to disparities in how frequently children hear cardinal number in everyday speech.

## Introduction

Multiple studies indicate that mathematical and number-specific language input from both parents and teachers predicts young children’s number word acquisition (Huttenlocher et al., 1991; Goodman et al., 2008; Levine et al., 2010), preschool mathematical knowledge (Klibanoff et al., 2006; Susperreguy and Davis-Kean, 2016; Casey et al., 2018), as well as grade school arithmetic ability (Case and Griffin, 1990; Jordan et al., 2009; Niklas and Schneider, 2014; Casey et al., 2018). For example, Susperreguy and Davis-Kean (2016) found that the frequency of math talk between mothers and preschoolers was positively related to children’s kindergarten mathematical ability 1 year later. As a whole, these results indicate that children acquire number words and concepts earlier and more readily the more frequently they hear them. Given the robust and well-documented cross-cultural differences in mathematical achievement between Mandarin Chinese and English-speaking grade school children (e.g., Mullis et al., 2016), previous research has examined the amount and types of numeric language input Mandarin and English-speaking children receive from their caregivers prior to entering formal schooling. Chang et al. (2011) found that Mandarin speaking Chinese parents use significantly more number-specific language than their English-speaking counterparts when interacting with their preschool-aged children in naturalistic settings. Although differences between the Chinese and English languages mightcertainly contribute to these striking differences in the amount of number talk in Mandarin vs. English, it is difficult to ascertain whether parental number speech is more greatly affected by language or parent level factors in cross-national populations, where language and cultural contexts are closely intertwined. Thus, the current research seeks to control for parent-level influences, such as differences in mathematics education or beliefs about the value of mathematics, by examining whether Mandarin-English bilingual speaking parents vary the frequency of number talk when providing speaking in Mandarin vs. English.

Prior to formal schooling, language input has been shown to be an important source of informal learning for young children. A direct relationship has been documented between child vocabulary acquisition and the overall amount of parental language spoken to children during daily activities (e.g., Huttenlocher et al., 1991; Goodman et al., 2008). More specifically, the types and tokens that appear in parents’ speech to children appear to influence the words earliest acquired in children’s vocabularies. For instance, the more often a particular verb is used in parents’ speech, the more often that same verb appears in children’s speech in subsequent weeks (Naigles and Hoff-Ginsberg, 1998). Although most input studies have suggested that breadth of vocabulary is directly related to input frequency, it is also noteworthy that depth of vocabulary, or understanding of words and their meanings, is also strongly related to frequency of input (Vermeer, 2001).

Given the strong relationship between parental language input and children’s acquisition, an understanding of the type of numeric language children hear is critical for understanding children’s numerical development. Although few studies have examined parental number speech specifically, previous findings strongly suggest that parents who frequently talk about number should have children who acquire numerical terms more readily and more deeply than children who hear number terms less often (cf. Vermeer, 2001). Such findings have strong implications for numeric input, and indeed, recent research (Levine et al., 2010) finds that children who hear more number words at 30 months of age say more number terms at 30 and 38 months of age. Additionally, the amount of teachers’ math-related talk is significantly correlated to the growth of preschooler’s mathematical knowledge over the course of a school year (Klibanoff et al., 2006). Levine et al. (2010) conducted a longitudinal study of 44 preschool children between the ages of 14 and 30 months. After controlling for socioeconomic status, they found that parental number talk predicted children’s understanding of cardinal numbers at 46 months. As a whole, these findings suggest that differences in numeric language input may result in different levels of numerical competence and thus may factor into the early differences between English-speaking and Mandarin-speaking children in tests of mathematical performance (e.g., Mullis et al., 2016). Although a majority of comparative research has focused on school-aged children, studies have shown that even prior to formal schooling, Chinese children outperform American children in mental addition (Geary et al., 1993). At the start of kindergarten, Chinese children, who had not yet received any formal education, showed a 3:1 advantage over their American counterparts on a paper-and-pencil test of addition. Siegler and Mu (2008) also found that Chinese kindergartners not only perform better than their American peers on arithmetic problems, but also on a novel mathematical task, number-line estimation.

Thus, a wide body of research has examined educational, cultural, as well as linguistic factors that might account for the disparities in mathematical performance between Mandarin and English-speaking children. This paper will focus on one additional factor: the structural and pragmatic differences between the two languages that may lead to divergence in the amount and type of number speech that parents use when interacting with their children.

In a cross-national, cross-linguistic examination of naturalistic monolingual Mandarin Chinese and English transcripts from the CHILDES database (MacWhinney and Snow, 1990; Chang et al., 2011) found several distinct differences in the number language used by Mandarin speaking parents and English-speaking parents when speaking to their preschool-aged children (mean age 23.4 months). First, Mandarin speaking parents talked about number more often than English speaking parents overall. This included all instances of number terms and questions or requests for quantities. For instance, specific set sizes (e.g., the phrase “four crayons”) were used over 2.5 times more often in Mandarin compared to English. The sheer amount of numeric language input that native Mandarin speaking children receive from their parents may contribute to earlier and deeper understanding and acquisition of number words and concepts.

Moreover, there were marked differences in the types of number constructions that were used between the two languages (Chang et al., 2011). Although “one” was the most frequent number term in both languages, English speaking parents most often used “one” as a pronoun (e.g., “that one”), whereas Mandarin speaking parents primarily used “one” as a modifier (e.g., “one dog”). Indeed, this same pattern was found across all number terms. Pronouns were the most frequent type of number utterance found in the English-speaking sample, though pronominal use of number was found significantly less often in the Mandarin speaking sample. Mandarin speaking parents used numbers in *cardinal* constructions (e.g., “liang3^1^ zhi1 lao2 hu3,” or “two tigers”) to directly quantify sets of objects most often and did so significantly more often than English speaking parents. As cardinal numbers point to specific quantities, these types of constructions may guide young children’s attention to the concept of set size, or the cardinal number principle, while giving them more practice with quantities than their English-speaking peers. Comparatively, the pronominal use of “one” commonly found in English may provide vague information about quantity but does not necessarily emphasize set size when used in this manner (i.e., as a substitute for a noun). For example, when a parent refers to crayons when asking a child “Which *one* is red?” the focus is not on the “*oneness*” of each crayon, but rather its “*redness*,” or lack thereof. Thus, Mandarin speaking children, who more commonly hear numbers terms in a cardinal construction (e.g., “*one* kitten”), should have an easier time interpreting the number “one” as a quantity. This idea is in line with previous findings that suggest children better learn the meanings of novel adjectives (in the current case, a quantifier) when they are directly preceding a noun (Mintz and Gleitman, 2002).

Finally, a number of studies have also documented differences in the use of classifiers in languages like Mandarin and English (e.g., Cheng and Sybesma, 1998). Classifiers, or measure words (e.g., “slice” or “sheet” in English), were found in the majority of cardinal number utterances in Mandarin, but were essentially absent across all English transcripts. English count nouns, which have regular singular and plural forms (e.g., dog and dogs), do not typically take classifiers when quantified (i.e., one dog and two dogs). However, classifiers are required with almost all Chinese nouns, with rare exceptions, whether singular or plural (Chien et al., 2003). For example, *yi1 zhi1 gou3* means “one (unit) dog,” and *liang3 zhi1 gou3* means “two (unit) dog(s)” in Mandarin, where *zhi1* is a specific classifier that indicates an animal. The common pairing of classifiers with cardinal numbers could aid Mandarin speaking children in number learning, as the existence of a classifier may indicate to a child that a quantity is being enumerated, even if the child is unsure as to its exact meaning. For example, upon hearing “jiu3 zhi1 gou3” a Mandarin speaking child who has not encountered the word “jiu3” before can infer from linguistic context that “jiu3” indicates quantity because of the presence of the classifier. In contrast, hearing the phrase “nine dogs” does not provide a linguistic context that indicates that “nine” is a quantity.

Beyond structural differences between the English and Chinese languages, there are also cultural factors that may affect the ways Chinese and American parents talk to their children about number. Cultural differences in how education is viewed – particularly the study of mathematics – have been suggested as an explanation for the difference in mathematical achievement between school-aged children of the United States and China. Past research has indicated that Chinese parents typically place greater value on education than American parents. Chinese parents tend to set higher standards for their children’s academic achievement and have a greater tendency to share the responsibility for their children’s academic progress (Stevenson and Stigler, 1992). For example, Chinese parents spend a greater amount of time in helping their children with homework than do their American counterparts. Stigler et al. (1986) have stated that these differences may be due to the fact that Chinese parents emphasize the effect of effort on academic performance, whereas American parents emphasize innate ability. However, in Chinese culture, the years before first grade are considered an “age of innocence,” when academic progress is not a concern for youngsters (Stevenson and Stigler, 1992). This is in stark contrast to the United States, where parents spend hundreds of millions of dollars yearly on products that purportedly help very young children learn basic words and concepts (Healey et al., 2019). Given these robust cross-cultural differences in attitudes, how might a bicultural Chinese-American parent living in the United States interact with his or her young child regarding concepts such as number?

Although previous results (Chang et al., 2011) indicated definite differences in the types of number constructions Mandarin and English-speaking parents use when talking to their children about number, the naturalistic methods used to examine parental speech, as well as the nature of the transcripts analyzed did not allow careful examination of the factors underlying the differences in parental input. More specifically, language and culture could not be uncoupled in each language group, because the observations transcribed were taken of monolingual speakers in different countries, and cultural contexts. Thus, the individual influences of either language or culture alone on parent number speech could not be determined. Furthermore, the methodology used did not allow the original observers to control for the stimuli provided to each parent-child pair. Are differences in number usage more strongly influenced by language or culture? How do fluent bilingual speaking Chinese-American parents talk to their children about number in each language? The results of the present investigation may provide insight into whether math achievement differences seen between Mandarin speaking Chinese children and English-speaking American children might be more of a linguistic or cultural construct. If language has a larger influence, we might see that bilingual speakers behave differently (e.g., like a Mandarin monolingual vs. an English monolingual) depending on the language they use. However, if culture has a larger impact the way parents talk to their children, we might see less robust cross-linguistic differences within subjects.

The current study focuses on the influences of language on parental numeric input, but controls for language as well as culture. In this study, Mandarin-English bilingual speaking parents and their preschool-aged children were recruited to serve as participants. All participants were residents of Southern California and were proficient in both languages. Because a within-subjects design was used – the same subjects participated in both languages of interest – culture was kept constant. Each parent spoke in both English and Mandarin, using each language for half of the experimental session, providing equivalent control groups for each language, which was not possible in a cross-national design. All recruited parents lived and worked in the Los Angeles and San Gabriel Valley areas, and all children were enrolled in English speaking daycare or preschool programs. As we were also interested in cultural beliefs and practices, a survey was also given to assess the attitudes of bilingual, bicultural parents on math and education.

## Materials and Methods

### Participants

Twenty-two Mandarin Chinese-English bilingual parent-child dyads volunteered to participate in this study. The participants consisted of 11 male and 11 female preschool-aged children (mean age 49.14 months, *SD* = 10.60 months, range 29–69 months), 20 mothers, and two fathers. The mean age of parent participants was 38.36 years (*SD* = 4.02 years, range 31–49 years). At the time of testing, none of the children had yet to enter kindergarten. Parents and children were recruited from preschools, childcare centers, and Chinese language schools in Los Angeles and San Gabriel Valley area communities.

Participants were considered eligible for the study if the parent self-reported “fair” to “native-like” abilities in speaking and listening in both Mandarin Chinese and English on the Language History Questionnaire (Li et al., 2006). The Language History Questionnaire required parents to self-report their native language and all other languages that they knew, and to rate their proficiency in reading, writing, speaking, and listening in each language on a seven-point Likert-type scale (1 = very poor, 7 = native-like). Parents also provided the age at which they were first exposed to each foreign language, number of years spent learning each language, and rated their strength of accent in each language on a seven-point scale (1 = no accent, 7 = very strong accent). Parents also indicated the amount of time spent using each of their languages per day; with whom they used their languages to communicate; and during which activities they used each language. Frequency and type of language mixing was also described, as well as their preferences in language usage.

Twenty-one parents identified as native Mandarin speakers, and one identified as a native Cantonese speaker who self-reported “good” speaking and listening Mandarin abilities on the LHQ. All parents reported English as their second language. Parent’s self-reported “good” English spoken fluency (mean rating = 4.91, *SD* = 1.02, range 3–7) as well as “good” English listening fluency (mean rating = 5.09, *SD* = 1.11, range 3–7). All parents regularly spoke English outside the home.

Overall parents had a high level of education. Seven parents reported completing bachelor’s degrees, seven completed master’s degrees, four completed Ph.D. degrees, one earned a professional law degree (J.D.), and one earned an associate degree (A.A.). Two parents did not report their attained level of education. Fourteen of the parents were natives of Taiwan, R.O.C., six reported that they originated from the People’s Republic of China, and two were from Hong Kong, China. On average, parents had lived in the United States for 14.24 years (*SD* = 7.29 years, range = 3–26 years), and began learning English at 12.59 years of age (*SD* = 2.68 years, range = 5–20 years of age). Participants were tested in the laboratory, at their preschool or childcare center, or in their homes. The research protocol was approved by the UCLA Institutional Review Board. All parents provided written consent prior to participating in the study with their children.

### Procedure

Mandarin-English speaking bilingual researchers conducted this experiment. Equal numbers of subject pairs were randomly assigned to a specific order of stimuli and order of languages (Mandarin first, or English first) prior to participation. Researchers conversed with the subject pair in the assigned first language during the informed consent process and throughout the beginning of the session. The entire session was recorded on a digital camcorder.

Parents and children first participated in the picture books task. The child then completed three tasks intended to assess children’s counting ability and numerical knowledge: first the How Many? task, followed by the Give a Number task, and finally, the Number Comparison task. During the child’s participation in these counting tasks, parents completed Child Language and Educational Attitudes Questionnaire.

### Picture Books

Forty pages of full-color photographs depicting familiar objects were chosen as the stimuli for this experiment. Each stimulus appeared as an approximately 4 in by 5 in (10.16 cm by 12.70 cm) color photograph centered on an 8.5 in by 11 in (21.59 cm by 27.94 cm) sheet of white matte presentation paper. Objects were chosen to be familiar to both parents and children (e.g., common animals, toys, and household items) and to be readily identifiable in both Mandarin and English. Twenty of these photographs were selected because they depicted good examples of cardinal number situations (easily quantifiable sets of objects, e.g., nine crayons) that did not require the use of a classifier (e.g., slice or sheet) in English. The remaining 20 photographs were selected because they depicted good examples of settings where an English classifier could be used when labeling quantities. Classifiers are nouns that indicate a unit of measurement (e.g., two glasses of water, liang3 bei1 shui3) when labeling quantities. Fluent bilingual speakers confirmed that these 20 objects typically co-occurred with a classifying noun in both Mandarin and English. Each photograph included a different number of objects, ranging from one to ten. Four instances of each quantity (two each in cardinal stimuli, and two each in classifier stimuli) appeared in the set of 40 stimuli (i.e., four photos that depicting one object, four photos that depicted two objects, etc.). In order to minimize demand characteristics, stimuli were chosen such that they depicted several dimensions (including number) so that parents could discuss other attributes than number if they chose, such as color and shape.

The 40 pages were counterbalanced for order and divided into two books of 20 pages each (one book for each language) for each subject. This process was repeated three additional times to create a total of four counterbalanced orders. Across the four orders, each photograph appeared an equal number of times in the first half and the second half, and never appeared more than once in each counterbalanced order. Orders were created to allow opportunities to use English classifiers for half of each set of pictures.

Parents were handed the first book and were asked to discuss the pictures in the assigned first language with their child as if they were looking at a picture book at home. Parents were requested to avoid code mixing and parents were informed that they would be asked to switch languages later in the study. Participants were not timed and were allowed to talk about the pictures for as long as they chose to.

After viewing and discussing the first set of 20 pictures in the assigned first language, children were given a 5-min play break during which they played with toys such as Play-Doh or stuffed animals. Parents were asked to continue playing with their child while using the first assigned language as if they were doing so at home. Approximately 2 min and 30 s into the play period, parents were asked to switch into the second assigned language, and to continue playing while speaking the second language. This break allowed children a rest period from looking at the photographs and was further used as parent speech samples in each language, to assess parental fluency. Finally, the break served as a transition period in which subjects became accustomed to speaking and listening in the second assigned language.

Parents and children were next presented with the second book. Parents were asked to discuss the pictures in the second assigned language with their child, as would at home. As before, participants were not timed and were allowed to talk about the pictures for as long as they chose to.

### How Many Task

The materials used in the “How many?” task were familiar shapes (e.g., stars and apples) presented in a horizontal row. Four set sizes (10, 8, 7, and 6) were used. These quantities were chosen to ascertain each child’s counting ability. Close quantities (6 and 7; 8 and 10) were chosen to examine whether each child could correctly count each set of objects.

In the first assigned language, each child was asked to count two rows of objects aloud one at a time. That is, after counting one row of objects, the child was next asked to count a second row of objects. Next, in the second assigned language, each child was asked to count the remaining two rows of objects aloud one at a time. During the task, children typically had one attempt to count each row of objects, but children were asked to switch languages if they began to count in the wrong language and were asked to restart if their first counting attempt was inaudible.

### Give a Number/Counting Bears Task

In the “Give a Number/Counting Bears” task, a set of plastic counting bears and a paper plate were used. The experimenter placed 12 plastic bears of the same color in front of the child, and asked the child, in the first assigned language, to place three bears onto a paper plate. The experimenter then took the bears off of the plate, and asked the child, again in the first assigned language, to place five bears onto the plate. The experimenter then repeated this process in the other language, first asking the child to place five bears onto the plate, and then three bears.

### Number Comparison Task

In the comparison task, 4.25 in by 5.5 in (10.80 cm by 13.97 cm) index cards were presented with stickers on them. Each card displayed four, five, seven, or nine star-shaped or smiley face stickers.

#### First Assigned Language

Two cards were placed side by side in front of the child, one card with four stickers and one card with five stickers. Speaking in the first assigned language, the experimenter asked the child to point to the card with four stickers on it. The experimenter then removed the cards and presented two new cards – one card with seven stickers and card with nine stickers. Speaking in the first assigned language, the experimenter asked the child to point to the card with seven stickers. After the child pointed, these cards were removed.

#### Second Assigned Language

The cards with four and five stickers were placed back in front of the child, in the opposite positions that they were previously placed (e.g., if the card with four stickers was previously placed to the child’s right, it would then be placed on the child’s left during the second showing). Speaking in the second assigned language, the experimenter asked the child to point to the card with five stickers on it. This process was repeated in the second assigned language with the cards with seven and nine stickers on them, and the child was asked to point to the card with nine stickers on it.

### Child Language and Educational Attitudes Questionnaire

We designed and administered a questionnaire for parents to report their child’s language history, fluency, usage, and exposure to each language. Parents reported the child’s native, second, and any other languages; the age and context in which the child learned his/her second language, and the child’s speaking and listening ability in each language. A seven-point Likert type scale was used to rate language ability (1 = very poor, 7 = native-like). Parents also indicated the primary language they used with their child at home, and the percentage of time this language was used. They also reported the primary language the child used at home, and the percentage of time the language was used. Parents were also asked to report their child’s reading and writing abilities, if any, in each language, and to describe any language studies the child participated outside the home. Finally, parents were asked their opinions on academic achievement and extracurricular activities. A seven-point scale was used (1 = strongly disagree, 7 = strongly agree) to describe the parent’s level of agreement with a series of statements about their child’s future academic achievements (e.g., “My child will pursue post-graduate education”), as well as their opinions on extracurricular involvement, and development of skills and talents (e.g., “Extracurricular activities are as important as academics,” “Skills in areas such as mathematics or the arts are innate”). The child language and education survey used in this study is presented in its entirety in the Supplementary Material.

### Data Analysis

Recordings of the experimental sessions were examined for number-related speech. “Number utterances” were defined as speech that included a number term (e.g., one, two, first, and second) or a counting question or request, (e.g., “How many are there?” “Can you count these?”) Mandarin-English bilingual coders viewed the recordings, identified and categorized all number utterances, selected all number utterances that occurred with each stimulus, recorded the utterance, and noted its speaker. Interrater agreement between coders across over 20% of the recordings was 94.03%. Number utterances were also coded for the following six types of utterances.

#### Cardinal

Cardinal utterances included specific references to quantity when describing an object (e.g., one hat, yi1 ding3 mao4 zi) or a set of objects (six ducklings, liu4 zhi1 xiao3 ya1 zi).

#### Counting Routine

Counting routines occurred when the child or parent counted objects without specifically labeling them (e.g., “one, two, three, and four,” “yi1 er4 san1 si4”). These sequences typically occurred after parents asked *counting questions*, which were also identified and recorded.

#### Counting Questions

Counting questions occurred when the parent asked the child to count a set of objects or to otherwise indicate a quantity (e.g., “How many pencils are there?” “Can you count the puppies?” “You3 ji3 ge4 qian1 bi2?” “Shu3 yi1 shu3 kan4 you3 ji3 ge4 gou3 gou”).

#### Pronoun

Pronoun usage, or the use of a number term without a direct cardinal referent in cases where the number term could be grammatically replaced with a noun (e.g., this *one*, those *two*, zhe4 *yi1* ge4, na4 *liang3* ge4), was also noted.

#### Idiom

Idiomatic usage of number terms, particularly the number one (yi1), which occurs regularly in Mandarin, was also identified and categorized. These types of utterances typically occurred when parents compared objects within pictures and declared them “the same” or “one type,” or “yi1 yang4” (e.g., “zhe4 liang3 zhi1 gou3 xiang4 ni3 de yi1 yang4,” which translates to “these two dogs are the same as yours”).

#### Other

Other documented categories of number speech that occurred rarely during this experiment included references to money (e.g., $4.25), age (e.g., 1 year old), and other number utterances that did not fall into one of the previously mentioned categories.

## Results

There were no differences in the amount of time parents spent “reading” the picture book in Mandarin vs. English. Across both languages, parents talked about number an average of 42.36 times (*SD* = 43.97, range 0–154). When speaking Mandarin, parents made an average of 23.82 number utterances (*SD* = 28.22, range 0–114). When speaking English, the same parents made an average of 18.59 number utterances (*SD* = 18.66, range 0–75). There did not appear to be any particular stimuli that generally elicited significantly greater or fewer number utterances than other stimuli in either or both languages. A paired-samples *t*-test did not reveal significant differences in the amount of overall parental number speech between languages.

### Pronoun Versus Non-pronoun Utterances

Parents used number terms in several ways in their interactions with their children. One of these forms was the pronominal form, which occurred when number terms such as “one” were used in place of a noun (e.g., “Do you like this *one*?” “Ni3 xi3 huan1 zhe4 yi1 ge4 ma?”) without affecting the grammaticality or semantics of the sentence. That is, the parent could have replaced the number term with a noun such as “dog,” or “(zhi1) gou3,” without affecting the meaning of their speech. Pronominal number utterances were coded separately from non-pronoun usages of number, which were defined as the use of a number term that could not be grammatically replaced with another noun. Pronoun and non-pronoun forms of number statements were examined separately because they express slightly different meanings. For example, “this one is red” and “this crayon is red” share similar meanings, specifically that red is a property of a crayon. In these phrases, the number term does not serve as an explicit quantifier. The example “this one is red” would have been coded as a pronoun utterance in the present study. On the other hand, “there is one red crayon” emphasizes the quantity of crayons that are red and would have been coded as a cardinal number utterance. Both “one” and “red” in this example act as descriptors of the crayon, with “one” serving as a quantifier.

In general, parents made fewer pronoun number utterances when they were speaking Mandarin (*M* = 4.14, *SD* = 5.46) compared to when they were speaking English (*M* = 7.59 *SD* = 8.08). Pronoun number utterances made up 40.39% of parental number speech in English, but only 17.5% of parental number speech in Mandarin. A paired-samples *t*-test showed a marginal trend suggesting that pronoun number utterances are made more often when parents speak English (compared to when parents speak Mandarin, *t*(21) = 2.01, *p* = 0.058.

**FIGURE 1:**
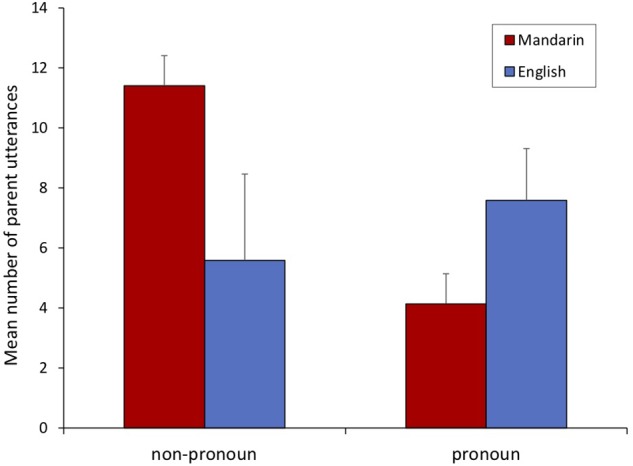
Mean number of pronoun and non-pronoun number utterances when parents spoke in Mandarin and English. Error bars indicate standard error of the mean.

Between Mandarin and English, the amount of non-pronoun number speech differed substantially. Figure 1 presents the average number of pronoun and non-pronoun number utterances spoken by parents in both languages. When speaking Mandarin, parents used a greater amount of non-pronoun number speech (*M* = 11.41, *SD* = 16.17) than when speaking English (*M* = 5.59, *SD* = 13.48). A paired-samples *t*-test revealed significant language differences, *t*(21) = 2.43, *p* = 0.024.

### Other Number Categories

Idiomatic phrases containing number were significantly more common in Mandarin, and parents tended to use cardinal numbers and ask counting questions more frequently in Mandarin as well. There were no significant differences in the frequency of counting routines between languages.

### Classifiers

In a majority of Mandarin number speech, parents used classifiers, modifiers indicating units of measure (e.g., “slices” in seven *slices* of pizza, or “tiao2”in “jiu3 tiao2 ku4 zi”), when discussing photo stimuli with their children. Classifiers were used in 69.39% of all Mandarin number utterances. On the other hand, classifiers were used in English extremely rarely – only five times overall. These comprised only 1.22% of all English parental number utterances. A paired-samples *t*-test revealed significant language differences, such that parents made many more number utterances using classifiers when speaking Mandarin compared to when speaking English, *t*(21) = 11.296, *p* < 0.01.

### Counting Tasks

**FIGURE 2:**
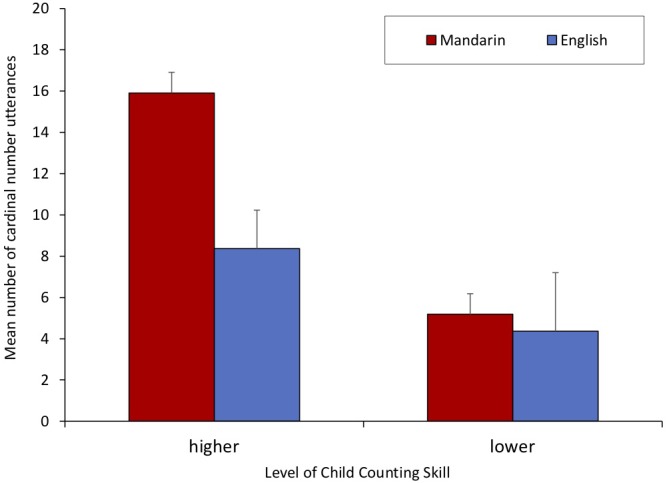
Mean number of parents’ cardinal number utterances by level of child counting skill. Errors bars indicate standard error of mean.

Children were awarded one “point” for correct completion of each of the following ten counting tasks: counting in English and Mandarin, giving three and five objects in both languages, as well as choosing between four and five, and seven and nine in both languages. Scores ranged from 1 to 10, with a mean score of 6.18 (Median = 6.50, *SD* = 3.22). Children with scores higher than 6.50 (the median score) were considered “higher skilled,” and children with scores below the median were considered “lower skilled.” Eleven children fell into each category. The average age of the children in the higher skilled group was 56.36 months (Median = 58 months, *SD* = 7.76), whereas the average age of children in the lower skilled group was 41.91 months (Median = 42 months, *SD* = 7.80). To determine whether amount of parental number speech differed as a function of child counting skill, 2 × 2 within-subjects ANOVAs compared parental number language input across languages and levels of number skill. Analyses revealed a marginal trend of parents speaking a greater amount of cardinal number terms when discussing stimuli with higher number skills (*M* = 12.14, *SD* = 9.15) compared to when they spoke to children with lower number skills (*M* = 4.77, *SD* = 8.01), *F*(1,21) = 3.88, *p* = 0.077. As shown in Figure 2, a significant interaction was found between language and number skill when parents used number in cardinal forms while discussing number stimuli, such that children were most likely to receive Mandarin number speech in these categories if they were more highly skilled in counting, *F*(1,21) = 5.038, *p* < 0.05. However, because older children had higher number skills and younger children had lower number skills, it was not possible to disentangle whether vocabulary or age affected the amount of cardinal number utterances that parents provided to children. Thus, conclusions about the relation between children’s number skills and parents’ cardinal number use are limited due to the correlations between age and counting skills in the present sample.

### Survey Data

According to parent reports, all but one child participant in the current study learned Mandarin as a native language. Four of the children also learned another language or dialect simultaneously since birth, two learned English along with Mandarin, one learned Cantonese, and one learned Taiwanese. One participant spoke English as a native language but began learning Mandarin as his second language at home from his mother at age 2 years. The 19 child participants who did not speak English as one of their native languages all began learning English as their second language at an average of 23.38 months of age (*SD* = 12.10). All but one child learned their second language either at home, daycare, or preschool. The remaining child learned English, according to parental report, from television.

Parents were asked to rate their child’s language speaking and listening fluencies on a one to seven scale, with one being “very poor,” four being “functional,” and seven being “native-like.” The average first language (L1) speaking fluency rating was 5.16 (between “good” and “very good,” *SD* = 1.30). The average L1 listening fluency rating was 5.74 (*SD* = 1.16). One parent did not report a listening fluency. Parents rated their children’s second language (L2) speaking fluency at an average of 4.29 (*SD* = 1.56), and their L2 listening fluency at an average of 4.69 (*SD* = 1.33). Again, the same parent did not report a listening fluency for L2.

Twenty of the 22 participating parents (90.91%) spoke Mandarin to their children a majority (>50%) of the time. Parents reported that they used Mandarin when speaking to their children an average of 80% of the time (*SD* = 19%). One spoke to their child in both Mandarin and English, using each language 50% of the time, and one spoke Cantonese to their child 50% of the time. Parents also reported that a majority of the children (17 children, or 77.27%) used Mandarin the majority of the time when speaking to them at home. Four children (18.18%) spoke English to their parents a majority of the time, and one used mostly Cantonese at home with his mother. On average, children used their “majority” language about 69% of the time (*SD* = 22.10%).

**FIGURE 3:**
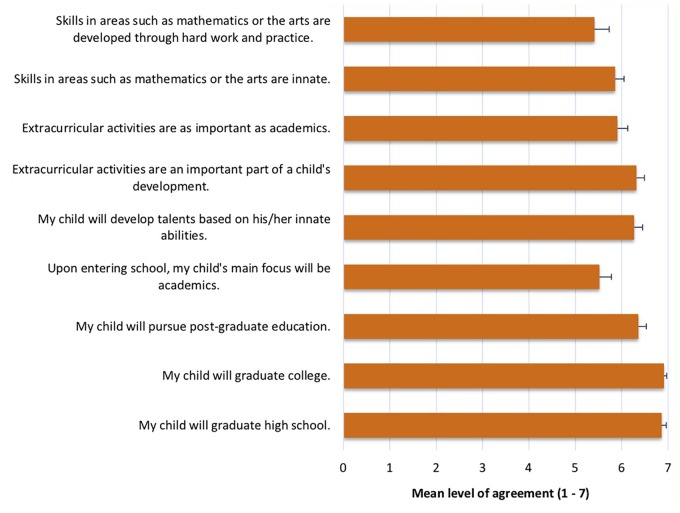
List of statements and mean ratings of participant answers on parent education survey. Error bars indicate standard error of the mean.

A majority of the parents (17 or 77.27%) reported that they either had enrolled their child or planned to enroll their child in extracurricular activities (e.g., music, art, and sports). The remainder of the survey asked parents to rate their level of agreement on a one to seven Likert scale (one being “strongly disagree,” four being “neither agree nor disagree,” and seven being “strongly agree”) on nine statements regarding their opinions on education, extracurricular activities, and innate vs. acquired skills. In general, parents’ levels of agreement on all statements (e.g., “My child will graduate high school,” “Extracurricular activities are an important part of a child’s development,” and “Skills in areas such as mathematics or the arts are innate”) were high (mean ratings ranged from 5.41 to 6.91), with little variation across subjects (SDs ranged from 0.29 to 1.21). Figure 3 displays all nine statements on the survey and their mean ratings. Although we had hoped to examine how parents’ attitudes about education related to the amount that parents talked about number with their children, parental attitudes toward education were uniformly high, and there was little variation between parents.

### Fluency and Parental Numeric Input

In order to assess whether amount of parental number speech differed as a function of parental fluency in English, a median split was performed to create two groups based on self-reported parental speaking and listening fluency in English. Parents self-reported a median rating of “good” spoken and listening fluency in English on the LHQ (i.e., 5 on the 7-point Likert scale), with scores that ranged from “fair” (3) to “native-like” (7). Fourteen parents rated themselves “good” to “native-like” (i.e., 5–7 on the 7-point Likert scale) on English speaking and listening on the LHQ. These parents were considered the “higher English fluency” group. The remaining eight parents rated themselves “fair” or “functional” (i.e., 3 or 4 on the 7-point LHQ Likert scale, respectively) in English speaking and listening, and were considered the “lower English fluency” group.

Multiple independent samples *t*-tests were conducted to determine if fluency in English affected the amounts of parent number speech to children, comparing across all categories of number speech. No significant differences were found between parents who self-reported higher or lower English fluency in speaking and listening across all categories of number speech in both languages, both stimulus types, and all categories of number utterances analyzed in the study. There were also no trends within the data that suggested a higher or lower level of fluency in English affected the amounts of number speech parents made in any category. Parents, also, on average, tended to rate their child’s fluency in both languages within 1.5 or fewer points of their own fluency in speaking or listening of the same language. Thus, it is also unlikely that child language skill affected parental number speech.

## Discussion

The present study aimed to determine whether parental number speech to preschool aged children varies between Mandarin Chinese and English in bilingual speakers of both languages. Several key differences were found in the amount and frequency of number speech used between languages.

Similar to previous work (Chang et al., 2011), pronoun number utterances were made much more often in English than in Mandarin, in which they were relatively uncommon. This suggests that the similar trend among native monolingual speakers in English may not be based in cultural differences between English and Mandarin speakers residing in different countries, as the same result was found within these bilingual subjects when speaking English. Also, because pronominal usage of number terms does not emphasize the dimension of quantity as clearly as cardinal number constructions (“that one,” as opposed to “one shirt”), this finding suggests that even bilingual children receive less explicit numeric input in English compared to Mandarin. The prevalence of pronoun number speech in English and the greater amount of counting questions and cardinal constructions used in Mandarin suggests that preschoolers may have greater exposure to the concepts of cardinality, quantity, and number-related vocabulary words through Mandarin than through English.

Indeed, when examining the non-pronominal number utterances between the two languages, these utterances occurred significantly more often when parents spoke Mandarin. The two most commonly spoken non-pronominal number utterances were cardinal numbers and counting questions. Both of these types of number speech occurred more often when parents spoke Mandarin.

In a cardinal number statement, the number term is used as a descriptor, or adjective, which modifies the noun it is quantifying. Previous research has indicated that children are more likely to learn the meanings of novel adjectives when the words are used as modifiers for “strong” nouns with coherent category information (e.g., Mintz and Gleitman, 2002). In the present study, all objects were chosen as familiar members of coherent categories such as animals, food items, and household objects. In line with prior findings, the greater exposure to cardinal number statements that children receive when their parents speak Mandarin may help them learn the meanings of number terms when used in adjectival form to a much greater extent than when they hear number terms in a more indefinite form, such as the pronominal form commonly found in English number speech. Because true understanding of mathematical operations presupposes knowledge of the cardinal principle of number, the understanding of cardinality that may arise from Mandarin number input that bilingual children are receiving prior to formal schooling may in fact help them learn how to manipulate numbers earlier and with more ease than their monolingual English-speaking peers. Because the children in the present study interact with their parents at home mostly in Mandarin, it may be reasonable to infer that in everyday interaction with their parents, children receive many more cardinal number statements than pronominal number statements. This would not only suggest that their number-related Mandarin vocabulary and concept development may be more advanced than English monolingual children of the same age, but also supports the idea that these bilingual preschoolers may show similar mathematical advantages to the Chinese pre-kindergarteners in the Geary et al. (1993) study due to the numeric input they are receiving from their parents.

Although the stimuli presented in this study were carefully selected to allow parents to use classifiers in both languages, bilingual parents very rarely used classifiers when speaking English, however, bilingual parents used classifiers in the majority of their number utterances for both types of stimuli in Mandarin. Though it was impossible to design a set of stimuli that did not require classifier usage in Mandarin to create perfect controls for the “cardinal” set of stimuli in English, it is still surprising that less than 3% of the classifier stimuli presented in English (e.g., “nine pairs of pants,” “seven slices of pizza,” and “eight scoops of ice cream”) elicited classifier use. This result strongly suggests that numbers may be more salient in Chinese in part due to the omnipresence of necessary classifier usage when describing sets of objects, even if quantity is not the focus. For example, classifiers are used in Mandarin pronominal utterances with or without number terms (“yi1 ge4” translates to “one (unit),” while “zhe4 ge4” translates to “this one” without the usage of a Mandarin number term). Therefore, children may learn to be attentive to the idea that a classifier indicates a “unit,” which inherently implies numerosity, even in cases where specific quantities are not labeled. The constant usage of classifiers in Mandarin as an implicit or explicit indicator of cardinality may have a direct relationship with Mandarin speaking children’s development of the understanding of number.

Other distinctions between Mandarin and English may also contribute to differences in the amount of cardinal number usage. For example, the singular and plural markings in. language may influence how number words are learned (Sarnecka et al., 2007). Although English has a regular plural form that is used to indicate quantities larger than one (e.g., tiger vs. tigers) Mandarin nouns do not have a regular plural form, and thus may rely on specific cardinal numbers to denote plurality. Because the current study examined only one particular classifier language (Mandarin), it is unknown whether gains in cardinal number usage would also be observed in other classifier languages. Future studies should consider how a broader set of classifier languages affect the amount of cardinal number used in speech to children and how this interacts with specific language features and cultural influences to create number understanding in children.

As expected, younger children performed more poorly than older children when asked to count and manipulate numbers in both languages. Children who were less well versed in counting (mean age 3.5 years) tended to perform better when asked to count in English. Performance was similar across languages and counting tasks for the older, more highly skilled group (mean age 4.7 years). This suggests that the younger children are learning to count in English earlier than in Mandarin, although their counting seems to be more a result of rote memorization of counting numbers, rather than conceptualization of the correspondences between counting, sequence, and quantity. Younger children could often verbalize counting numbers, in order, from 1 to 10, but were unable to give the experimenter three or five objects.

Overall, the similarity in Mandarin number input between the bilingual sample in the present study and the Mandarin sample in the Chang et al. (2011) cross-national study suggests that parental number speech may not differ significantly based on the host culture in which the language is used. In the same way, the frequency of English pronominal number utterances was comparable between English monolingual speakers in the previous study and speakers of English as a second language in the present study. These results indicate that exposure to Mandarin Chinese during preschool ages provides children with more experience with direct quantification of objects and usage of classifiers, which imply cardinality (see Hornburg et al., 2018 for a discussion of relations between mathematical language and children’s understanding of cardinality). The amount and frequency of cardinal number utterances may help children to develop an earlier understanding of the cardinal principle of number than their monolingual English-speaking peers, who receive less input of this nature from their parents’ speech. A number of studies suggest that difficulties with mathematics in elementary school derive from weaknesses in basic number competencies (Malofeeva et al., 2004; Gersten et al., 2005; National Mathematics Advisory Panel, 2008; Jordan et al., 2009) and basic number competency is heavily affected by the amount of number input a child hears (Case and Griffin, 1990; Geary, 1995; Jordan et al., 2009; Levine et al., 2010). Indeed, programs that increase exposure to number words have led to significant gains in elementary school mathematics outcomes (Griffin et al., 1994; Aragón-Mendizábal et al., 2017). It is possible that in this same way, speaking or hearing more cardinal number language may contribute to the math competencies prior to entering formal schooling seen in Mandarin speaking children (Geary et al., 1993).

Due to the strong coupling between language and culture, it is impossible to rule out cultural influences completely as a cause for the cross-linguistic differences seen in bilingual parental numeric input, although the results do suggest that the usage and knowledge of Mandarin Chinese alone may positively affect parents’ number speech to their children. Regardless, numerous cross-cultural differences also contribute to early numeracy performance (see Cankaya and LeFevre, 2016 for review). Consequently, child speakers of Mandarin, whether monolingual or bilingual, may already differ from monolingual child speakers of English in their understanding of and their ability to count and manipulate numbers by the beginning of kindergarten, due to the disparate frequencies, amounts, and types of number language input they receive from everyday interactions with their caregivers. In this case, a preschool math advantage due to parental number input may then be an early contributor to later differences in mathematical achievement seen across cultures and languages throughout formal education.

## Ethics Statement

The research protocol was approved by the UCLA Institutional Review Board. In all instances, the participating parent was given information about the study and provided written consent for themselves and their child prior to beginning the study.

## Author Contributions

All authors listed have made a substantial, direct and intellectual contribution to the work, and approved it for publication.

## Conflict of Interest Statement

The authors declare that the research was conducted in the absence of any commercial or financial relationships that could be construed as a potential conflict of interest.

## Footnotes

1 Throughout this paper, *Hanyu pinyin*, a standardized Romanization system for Mandarin Chinese, is used to represent Chinese characters. The number next to each Romanized character denotes the tone (1–4) that is used to correctly pronounce the character in Mandarin.
