# Supplementary material for: How Bilingual Parents Talk to Children About Number in Mandarin and English
Source: Front Psychol. 2019 May 14;10:1090. doi: 10.3389/fpsyg.2019.01090 (PMC6527767; doi:10.3389/fpsyg.2019.01090)
Supplement: Supplementary file 1 [file Data_Sheet_1.docx]

Supplementary Material

How Bilingual Parents Talk to Children about Number in Mandarin and English

Alicia Chang*, Catherine M. Sandhofer*

*** Correspondence:** Alicia Chang: alicia.chang@gmail.com

# Parent Language and Education Survey

Please answer the following questions about your child:

Date of Birth: Sex: M / F

Birthplace (city, country):

Native, or first language spoken (if your child has always been fully bilingual, please specify):

Second language:

Age at which your child started to learn his/her second language:

Where (or from whom) has your child learned to speak his/her second language?

Please list all languages your child knows in order of most proficient to least proficient. Rate his/her ability on the following aspects in each language. Please rate according to the following scale (write down the number in the table):

very poor poor fair functional good very good native-like

1 _________ 2_________3_________4_________5_________6_________7_________

| Language | Speaking fluency | Listening ability |
| --- | --- | --- |
|  |  |  |
|  |  |  |
|  |  |  |
|  |  |  |
|  |  |  |

What language do you primarily speak to your child at home?

What percentage of the time do you use this language?

What language does your child primarily use when speaking to you at home?

What percentage of the time does he/she use this language?

Please describe other language usage between you and your child, or any other caregivers at home.

Does your child currently read and/or write? Please describe, and include the languages in which he/she reads and/or writes. If not applicable, please write N/A.

Do you or do you plan to enroll your child in language studies outside the home? If so, please describe, including the age at which you plan to enroll or enrolled your child, the languages studied, and the type of instruction you are interested in. If not applicable, please write N/A.

If your child is currently involved in extracurricular activities (e.g., music or sports), please list them here. If he/she is not, please list the age at which you might be interested in enrolling them in extracurricular activities, and the activities you might enroll them in. If not applicable, please write N/A.

Next to the following statements, please write the number that most closely corresponds to your opinion regarding the statement.

neither

strongly somewhat agree nor somewhat strongly

disagree disagree disagree disagree agree agree agree

1_________ 2_________3_________4_________5_________6_________7_________

My child will graduate high school. ______

My child will graduate college. ______

My child will pursue post-graduate education. ______

Upon entering school, my child’s main focus will be academics. _______

My child will develop talents based on his/her innate abilities. ______

Extracurricular activities are an important part of a child’s development. ______

Extracurricular activities are as important as academics. ______

Skills in areas such as mathematics or the arts are innate. ______

Skills in areas such as mathematics or the arts are developed through hard work and practice. _____
